# Dengue E Protein Domain III-Based DNA Immunisation Induces Strong Antibody Responses to All Four Viral Serotypes

**DOI:** 10.1371/journal.pntd.0003947

**Published:** 2015-07-28

**Authors:** Monica Poggianella, José L. Slon Campos, Kuan Rong Chan, Hwee Cheng Tan, Marco Bestagno, Eng Eong Ooi, Oscar R. Burrone

**Affiliations:** 1 International Centre for Genetic Engineering and Biotechnology, Trieste, Italy; 2 Program in Emerging Infectious Diseases, Duke-NUS Graduate Medical School, Singapore; Federal University of São Paulo, BRAZIL

## Abstract

Dengue virus (DENV) infection is a major emerging disease widely distributed throughout the tropical and subtropical regions of the world affecting several millions of people. Despite constants efforts, no specific treatment or effective vaccine is yet available. Here we show a novel design of a DNA immunisation strategy that resulted in the induction of strong antibody responses with high neutralisation titres in mice against all four viral serotypes. The immunogenic molecule is an engineered version of the domain III (DIII) of the virus E protein fused to the dimerising CH3 domain of the IgG immunoglobulin H chain. The DIII sequences were also codon-optimised for expression in mammalian cells. While DIII alone is very poorly secreted, the codon-optimised fusion protein is rightly expressed, folded and secreted at high levels, thus inducing strong antibody responses. Mice were immunised using gene-gun technology, an efficient way of intradermal delivery of the plasmid DNA, and the vaccine was able to induce neutralising titres against all serotypes. Additionally, all sera showed reactivity to a recombinant DIII version and the recombinant E protein produced and secreted from mammalian cells in a mono-biotinylated form when tested in a conformational ELISA. Sera were also highly reactive to infective viral particles in a virus-capture ELISA and specific for each serotype as revealed by the low cross-reactive and cross-neutralising activities. The serotype specific sera did not induce antibody dependent enhancement of infection (ADE) in non-homologous virus serotypes. A tetravalent immunisation protocol in mice showed induction of neutralising antibodies against all four dengue serotypes as well.

## Author Summary

Dengue disease is a mosquito-borne viral infection caused by Dengue virus (DENV), one of the most important human pathogens worldwide. DENV infection produces a systemic disease with a broad symptomatic spectrum ranging from mild febrile illness (Dengue Fever, DF) to severe haemorrhagic manifestations (Dengue Haemorrhagic fever and Dengue Shock Syndrome, DHF and DSS respectively). To date there is no vaccine available to prevent dengue disease. We show here a strategy of immunisation, tested in mice, that elicits a strong immune response against the four different DENV serotypes. The novelties presented in our work open the way to the development of an efficient vaccine accessible to developing countries.

## Introduction

Dengue is a mosquito-borne viral infection caused by dengue virus (DENV), one of the most important human pathogens worldwide [1]. The infection produces a systemic disease with a broad spectrum of outcomes, ranging from non-symptomatic/mild febrile illness (Dengue Fever, DF) to severe plasma leakage and haemorrhagic manifestations (Dengue Haemorrhagic Fever, DHF) that can further evolve into potentially fatal conditions (Dengue Shock Syndrome, DSS) [2, 3]. DENV, which is spread by *Aedes spp*. mosquitoes, is widely distributed throughout the tropical and subtropical regions of the world [2]. About 3 billion people, in over 100 countries, are estimated to be at risk of infection, with over 300 million infections, 500,000 episodes of DHF manifestations and 20,000 deaths reported each year [1, 4]. The remarkable spread and impact of the disease led the World Health Organization to classify it as the “most important mosquito-borne viral disease in the world” [5].

Four different serotypes of dengue viruses (DENV1, DENV2, DENV3 and DENV4) have been identified, all of which are pathogenic in humans [6]. Infection with any one serotype induces lifelong immunity against that specific serotype, with only transient cross-protection against the three other serotypes [7–9]. In fact, severe manifestations of dengue infection are generally associated with secondary infections involving different viral serotypes; this happens through a mechanism known as antibody-dependent enhancement of infection (ADE) [8, 10]. In ADE, recognition of viral particles by cross-reacting, but weakly or non-neutralising antibodies, leads to an increased Fc receptor-mediated uptake of immature or incompletely neutralised viruses by monocytes, macrophages, and dendritic cells, which represent the primary targets of dengue virus infections in humans, resulting in increased infectivity and deterioration of the patient’s clinical condition [11]. This is critical in dengue vaccine development, since an immunogen that does not elicit fully neutralising antibodies to all four serotypes may contribute to disease, rather than prevent infection [12]. Given the lack of efficient treatment against the infection and the risk to human health, in particular (but not only) in developing countries, research to develop an efficient vaccine has become an increasing but yet unsuccessful task.

DENV is an enveloped virus of the Flaviviridae family, with a single-stranded, positive-sense RNA genome of around 11 Kb that encodes 10 mature viral proteins [13, 14]. Among these, the envelope glycoprotein (E) is the major constituent of the viral membrane envelope, which plays essential roles during the endosomal-mediated virus internalisation, by promoting attachment to the host cell and fusion to the cellular membranes [15–17]. The E protein is formed by three structural domains (DI, DII and DIII), separated by a stem region from the two trans-membrane domains that anchor the protein to the virus membrane envelope [16, 18]. While DII plays a major role in E dimerisation and harbours the hydrophobic fusion loop, DIII is an Ig-like domain that has been implicated in binding to cellular receptors [16, 19, 20]. The mature infective viral particle has a relatively round, smooth surface with a highly ordered icosahedral scaffold formed by 180 E molecules, that are distributed in 30 rafts of 3 parallel dimers organized in a herringbone pattern [19, 21]. Additionally, E is the most important target of the antibody immune response during infection [8, 22]. Antibodies against the upper lateral surface of DIII have been described as the ones with high neutralising capacity; coincidently, this region shows also the highest variability among serotypes, which accounts for the high specificity of these antibodies [12, 23, 24]. Thus, DIII of protein E has been widely considered as the antigen of choice for vaccine development.

Genetic vaccination, based on the delivery into cells of the host of DNA or RNA constructs capable of expressing and secreting the encoded selected protein [25, 26], has been extensively tested and found to be highly effective in inducing immune responses against a wide range of pathogens and conditions [27]. It offers the possibility to raise virus-neutralising activity in a simple and effective way due to the very low costs of production and excellent stability, which can be critical when dealing with the conditions found in some developing countries [27, 28].

In this study we present four DNA constructs, each one encoding the DIII of a different DENV serotype that were engineered to enhance expression and secretion in mammalian cells and tested as genetic vaccines in mice. Our results indicate that they were able to drive strong neutralising responses against all four serotypes, with the potential of further development.

## Materials and Methods

### Cell lines and viruses

HEK293 and HEK293T/17 cells (ATCC, Rockville, MD, USA, numbers CRL-1573 and CRL-11268, respectively) were cultured in Dulbecco's modified Eagle's medium (DMEM, Life Technologies, Paisley, UK) supplemented with 10% heat-inactivated foetal calf serum (FCS) (Life Technologies), 50 μg/ml gentamycin and 2 mM L-glutamine. To select HEK293 stable clones, 0.4 mg/ml Geneticin (G418, Life Technologies) was added. Mouse myeloma Sp2/0-Ag14 cells (ATCC CRL-1581) were cultured in RPMI 1640 medium (Life Technologies) supplemented with 10% heat inactivated FCS, 50 μg/ml gentamycin, 2 mM L-glutamine, 1 mM sodium pyruvate. Sp2/0 stable clones were grown in selective media containing 0.4 mg/ml Geneticin. BHK-21 (ATCC CCL-10) and Vero (ATCC-CCL-81) cells were grown in DMEM medium supplemented with 10% heat inactivated FCS, 50 μg/ml gentamycin and 2 mM L-glutamine. Vero FM cells (Vero E6 derivate, kindly provided by Dr. Toni Rieger, BNI, Hamburg, Germany) were maintained in the same conditions with 1% non-essential amino acids. *Aedes albopictus* C6/36 cells (ATCC CRL-1660) were grown at 28°C in RPMI medium supplemented with 10% heat inactivated FCS, 50 μg/ml gentamycin, 2 mM L-glutamine, 1 mM sodium pyruvate and 1% non-essential amino acids. THP-1 human monocytic cells were grown in RPMI 1640 medium (Life Technologies) supplemented with 10% heat inactivated FCS.

DENV1 Hawaii A strain, DENV2 NGC strain, DENV3 3140/09 isolate and DENV4 TC25 strain (kindly provided by Dr. Alessandro Marcello, ICGEB, Trieste, Italy) were used for plaque reduction neutralisation test (PRNT). All DENV strains were propagated in Vero (DENV3 in Vero FM), BHK-21 and C6/36 cells in complete medium containing 2% heat inactivated FCS. Viral neutralisation titres were determined by plaque assay on Vero cells. Unless indicated differently, all cell cultures were grown at 37°C with 5% of CO_2_.

### Plasmid DNA constructs

Sequences coding for the envelope ectodomains were obtained from DENV1 Nauru Island strain (GenBank accession number U88535.1), DENV2 New Guinea C strain (GenBank accession number AF038403), DENV3 3H87 strain (GenBank accession number M93130), and DENV4 Dominica strain (GenBank accession numbers AF326573.1).

The original and codon optimised DIII sequences of all DENV serotypes were obtained as synthetic fragments in pUC57 vectors from GenScript (Piscataway, NJ, USA). Each DIII sequence was fused to an immunoglobulin leader sequence (sec) at the N-terminus [29] and to the SV5 tag (GKPIPNPLLGLD) [30] at the C-terminus. DIII-CH3 constructs contained, in addition, the human IgG heavy chain constant domain 3 (γCH3) downstream of the SV5 tag. Both DIII-SV5 and DIII-SV5-γCH3 were cloned into a pcDNA3 vector in which the neomycin resistance gene was deleted and in vector pVAX (Life Technologies). DENV3 sE (3sE) coding region was also obtained as a synthetic gene and cloned in the same vectors. The DIII aminoacidic sequences from all DENV serotypes are shown in S1 Fig.

The DIII-εCH4 constructs were obtained by replacing SV5-CH3 with the human εCH4 [31], followed by either the biotin acceptor peptide (BAP) sequence (GLNDIFEAQKIEWHE) [32, 33] to obtain the secretory biotinylated DIII-εCH4 molecule, or a glycosyl-phosphatidylinositol (GPI) anchor signal [34] to obtain the membrane-bound DIII-εCH4-GPI.

3sE was also engineered, fused to BAP and roTag [35] at the C-terminus and cloned into a bigenic vector containing the gene for a secretory *E*. *coli* biotin ligase [33]. From this construct, 3DI/DII-BAP-roTag was derived after deletion of the DIII domain.

An additional DIII-SV5-γCH3 construct for DENV4 TC25 strain, which contains 3 aminoacid changes with respect to the DENV4 Dominica strain DIII sequence (L357F, N360Y and N384D), was obtained by site-directed mutagenesis (QuikChange XL Site-Directed Mutagenesis Kit, Agilent Technologies, La Jolla, CA, USA) on the Dominica strain construct following the instructions of the manufacturer.

### Expression of recombinant dengue molecules

Transient transfections of HEK293T/17 cells and stable transfections of HEK293 cells were performed essentially as described by Sambrook *et al*. [36], using circular or linearized plasmids respectively.

HEK293T/17 cells were seeded in 6-well plates at 5x10^5^ cells/well density and transfected using standard calcium phosphate method with 0.5–5 μg of plasmids. 24 h after transfection the culture medium was replaced with a serum-free medium, when required supplemented with 100 μM biotin (Sigma-Aldrich, St. Louis, MO, USA), and after 24 more hours cellular extracts and supernatants from transiently transfected cells were collected. To remove free biotin, the supernatants of samples containing biotinylated molecules were extensively dialyzed against PBS. Cellular extracts were prepared in 100 μl of TNN lysis buffer (100 mM Tris-HCl, pH 8, 250 mM NaCl, 0.5% NP-40) at 4°C, supplemented with Protease Inhibitor Cocktail (Sigma) according to manufacturer's instructions. The expression and secretion of the recombinant dengue molecules was confirmed by western blot.

To produce large amounts of biotinylated antigens, recombinant biotinylated DIII-εCH4-BAP of all four serotypes, 3sE-BAP-roTag, 4sE-BAP-roTag and 3DIDII-BAP-roTag proteins were expressed in stably-transfected HEK293 cells. HEK293 cells were transfected with 15 μg of BglII-linearized DNA using calcium phosphate technique. Stably-transfected clones were screened by ELISA and secretion of biotinylated proteins confirmed by western blot. Supernatants from the selected clones were collected after 72 h of culture in serum-free medium supplemented with biotin and dialyzed against PBS. In experiments involving the use of denatured biotinylated DIII-εCH4-BAP, the dialyzed supernatants were denatured in presence of 0.5% SDS (Sigma-Aldrich) and 2.5% 2-Mercaptoethanol (Sigma-Aldrich) and boiled for 10 min. N-Ethylmaleimide (NEM, Sigma-Aldrich) was then added and samples were extensively dialyzed against PBS before using.

To generate cell lines expressing membrane-bound DIII, Sp2/0 cells were stably-transfected with recombinant DIII-εCH4-GPI proteins. Transfections were performed by electroporation as previously described [37]. Clones were analysed after staining with FITC-labelled anti-human IgE antibodies (KPL, Gaithersburg, MD, USA, 1:500 in PBS with 3% BSA) in a FACSCalibur flow cytometer (BD Biosciences, San Jose, CA, USA).

### Western blot

Samples of cell lysates and supernatants were separated by 10% SDS-PAGE gels in reducing conditions and then transferred onto polyvinylidene difluoride (PVDF) membranes (Millipore, Temecula, CA, USA). Membranes were blocked for 1 h with PBS with 5% Milk and 0.1% Tween-20, probed for 1h with an anti-SV5 monoclonal antibody (1:10,000 dilution) and incubated for 1h with HRP-linked goat antibodies anti-mouse IgG, (Jackson ImmunoResearch, Newmarket, UK, 1:5000). As loading controls mouse mAb anti-tubulin (clone DM1A, Millipore) and rabbit antibodies anti-actin (Sigma) were used. Signals were visualized by ECL (ThermoFisher-Pierce, Rockford, IL, USA).

### Animal immunisations

5–6 weeks old, female Balb/c mice were purchased from Harlan (Milan, Italy). All mice were immunised three times at fifteen days intervals (Days 1, 15 and 30) by biolistic delivery of 1 μm gold particles coated with 1 μg of DNA using Gene Gun technology (Bio-Rad, Hercules, CA, USA); blood samples were collected at days 45 and 60 by sub-mandibular puncture. In the case of the tetravalent formulation, each animal was vaccinated with 2 μg of DNA (two 1 μg shots applied at different body sites) following the same vaccination protocol. Serum samples were collected and stored at -20°C until use.

### ELISA

Mouse sera were tested on mono-biotinylated DIII-εCH4-BAP and sE-BAP-roTag proteins. The relative concentrations of biotinylated proteins collected from the stably transfected clones were normalized by western blot and comparable amounts of biotinylated protein were used in coating the ELISA plates. Nunc Maxi Sorp Immuno-Plates (ThermoFisher-Nunc, Roskilde, Denmark) were pre-coated with 100 μl/well of 5 μg/ml avidin (Sigma) in 50mM Na_2_CO_3_/NaHCO_3_ buffer pH 9.5 and incubated overnight at 4°C. Plates were washed in PBST buffer (0.05% Tween 20 in PBS pH 7.4), blocked with 1% BSA in PBST for 1 h 30 min. at RT, and second-coated with the dialyzed biotinylated-antigen diluted in PBS (100 μl/well), at 4°C overnight. After washing, different 100 μl dilutions of sera from immunised mice were added to plates and incubated for 2h at RT. After washing, 100 μl/well of HRP-linked goat antibodies anti-mouse IgG γ-chain (Jackson ImmunoResearch, 1:50000) were added and incubated for 1h at RT. The bound conjugate was detected using TMB substrate (Sigma) for 10 min. The reaction was stopped with H_2_SO_4_ 1M and measured at 450 nm (OD_450_) on a Bio-Rad iMark microplate reader. The anti-dengue IgG titres were determined as the reciprocal of the dilution at which the OD_450_ was 3 times higher than that of the negative control serum. Negative control sera obtained from animals immunised with a construct containing an irrelevant protein fused to γCH3 showed the same performance as pre-immune sera or sera from animals immunised with empty vector.

### Evaluation of anti-DIII specific antibody concentration

The concentration of anti-DIII specific antibodies in sera from vaccinated animals was determined by creating a calibration curve obtained by plotting the OD_450_ values from an ELISA on biotinylated 3sE with different amounts of a previously quantified and purified sample of anti-dengue envelope mAb 4G2 (Millipore). mAb 4G2 recognises a conformational epitope on the fusion loop of all DENV serotypes [38, 39]; in our case, the antibody reacts strongly against the 3sE protein with almost 100% avidity, and was therefore used to generate the calibration curve. OD_450_ resulting from different dilutions of each pool of sera on its homologous DIII were interpolated into this calibration curve to obtain approximate concentrations of specific anti-DIII antibodies in sera. The concentrations are reported as the arithmetic means ± standard deviations of all the dilutions with OD_450_ included within the calibration curve.

### Dengue virus-capture ELISA

Plates were coated with the immunoglobulin fraction from a human serum cross-reactive with all 4 serotypes (15 mg/ml in 50mM Na_2_CO_3_/NaHCO_3_ buffer pH 9.5) (kindly provided by Dr. Vivian Huerta, Centre for Genetic Engineering and Biotechnology (CIGB), Habana, Cuba) and incubated overnight at 4°C. Plates were washed, blocked and second-coated with 4x10^4^ PFUs/well of each viral serotype for 2h at RT. After washing, plates were incubated for 1h at 36°C with 100 μl/well of the different anti-DIII sera (diluted to a concentration of 100 ng/ml) or negative control sera at an equivalent dilution. mAb dengue 1–11 (AbD Serotec, Kidlington, U.K.), specific for DENV1 envelope protein (used at 1 μg/ml) and a DENV panreactive serum (a kind gift of Dr. Vivian Huerta, Centre for Genetic Engineering and Biotechnology (CIGB), Habana, Cuba) were used as positive controls. For detection, HRP-conjugated goat anti-mouse IgG γ-chain (Jackson ImmunoResearch) was used.

### Avidity assay

Serum avidity index was measured by a modified ELISA protocol with urea washes [40, 41]. Briefly, the different sera were tested at dilutions corresponding to an OD_450_ value of 0.6–0.8. After incubation with serum, plates were washed two times (3 min each) in PBST, with or without 6M urea, and incubated with secondary antibody as described above. The avidity index was calculated as the ratio between the OD_450_ obtained after the urea treatment and the OD_450_ without urea, multiplied by 100.

### Cytofluorimetry

Serotype-specific immune sera (diluted 1:1000 in PBS with 3% BSA) were incubated with the stable Sp2/0 DIII-εCH4-GPI transfectants followed by Alexa488-conjugated goat antibodies anti-mouse IgG (Jackson ImmunoResearch, 1:1000) and analysed in a FACSCalibur (BD Biosciences).

### Immunofluorescence

Vero cells were infected with DENV1, DENV2, DENV3 and DENV4 at multiplicity of infection (MOI) of 0.1. 36 h post-infection cells were fixed with 3.7% paraformaldehyde (PFA) in PBS for 20 min and quenched with 100 mM PBS glycine. After washing with PBS, cells were permeabilized with 1% Triton in PBS for 15 min and blocked with 0.1% BSA PBS-Tween 0.1% for 1h. Viruses in infected cells were detected using serotype specific mouse anti-DIII sera (dilution 1:50), mAb 4G2 (dilution 1:400) and mouse control sera followed by Alexa488-conjugated goat anti-mouse IgG (diluted 1:1000). Images were acquired using a Zeiss (Goettingen, Germany) LSM510 META microscope.

### Plaque reduction neutralisation test (PRNT)

PRNT was carried out on Vero cells seeded at a density of 160,000 cells/ well 24h before infection in 24 multi-well plates. De-complemented mouse sera samples (30 min. at 56°C) were 2 fold serially diluted from 1:12.5 to 1:400 in DMEM serum-free medium. Then an equal volume of DMEM-diluted dengue virus containing 50 PFU was added and incubated for 1.5h at 36°C in a final volume of 60 μl. Vero cells were then washed with DMEM serum-free media, infected in duplicate with 25 μl of the neutralisation mixture and incubated for 1h at 36°C. Afterwards, the viral inoculum was removed and cells were overlaid with 1 ml of DMEM with 2% FCS and 3% carboxymethylcellulose (Sigma). Plates were incubated at 36°C for 7–8 days depending on serotype (7 days for DENV2 and DENV3, 8 days for DENV1 and DENV4). After this period, cells were washed twice with PBS, fixed for 20 min. with PFA 3.7% and stained with 1% crystal violet for 30 min. Plaques were counted and percentage of plaque reduction against control serum was calculated. Neutralising antibody titres were expressed as the serum dilution yielding a 50% plaque number reduction (PRNT_50_).

### Antibody-dependent DENV infection in THP-1 cells

The ADE method used was as previously described [42]. Briefly, serial two-fold dilutions of each serotype-specific anti-DIII sera were incubated with the virus for 1 hour at 37°C before added to THP-1 cells at a MOI of 10. At 72 h after infection, the culture was clarified by centrifugation, and the infectious titer of dengue virus in the culture supernatant was quantified with plaque assay.

### Statistical analysis

All data shown were calculated from at least four independent experiments done in duplicate or triplicate. Except for the avidity data (showed in boxplots), all data are represented as arithmetic means ± standard deviations and were analysed using GraphPad Prism (version 6.0) software. Unpaired two-tailed *t* test was used to analyse sets of data between two groups. *P* values of <0.05 were considered significant.

### Ethics statement

All animal procedures were approved by the Italian Ministry of Health (Ministero della Salute) and the ICGEB Animal Welfare Board (protocol DGSAF0024706) in compliance to laws and policies established in the legislation D. L.vo 26/2014 of the Italian Government.

## Results

### 1. Secretion of an engineered DIII version

Activation of B cells for effective induction of antibody responses in the framework of DNA-based immunisations requires optimal antigen expression and secretion from the cells of the mammalian host [43].

**Fig 1:**
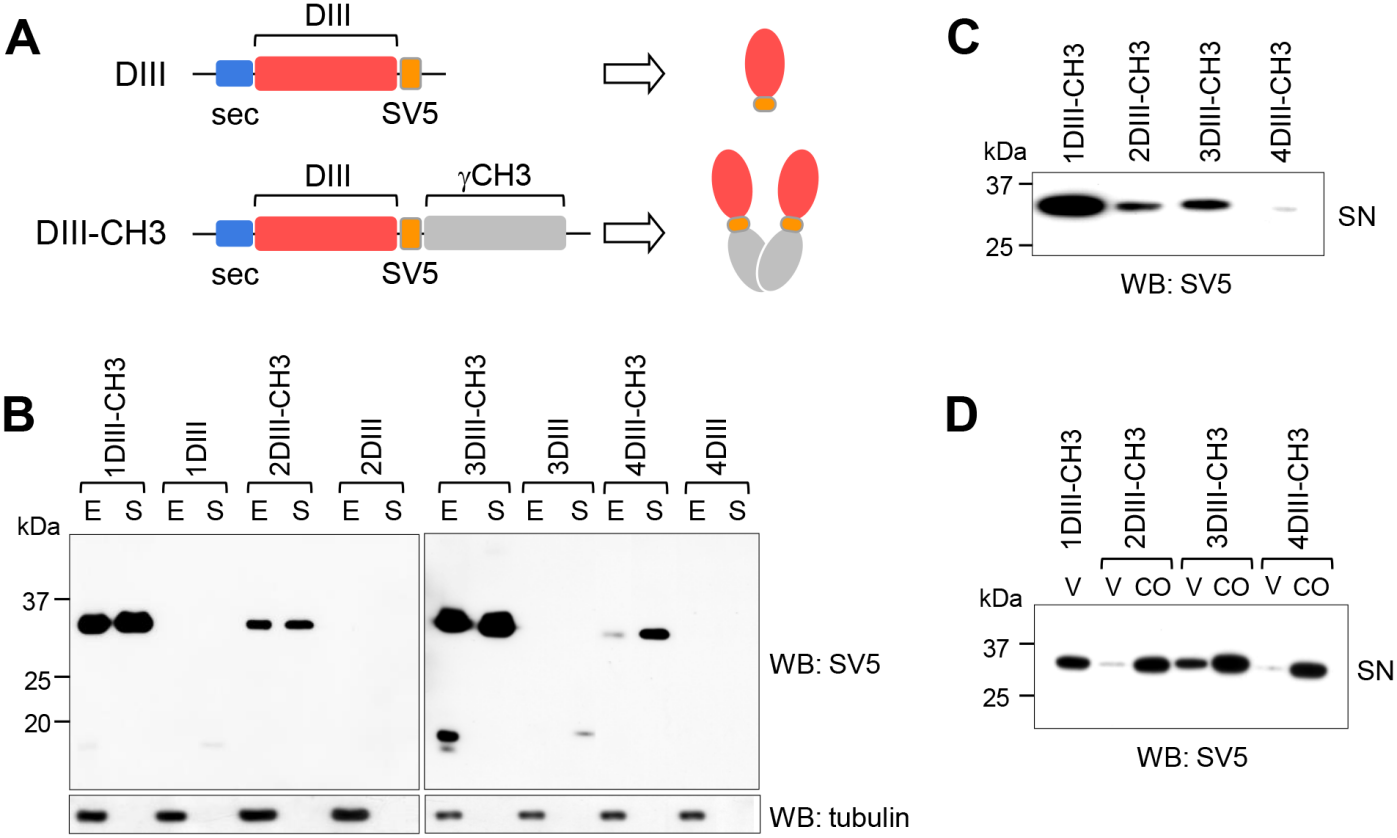
Optimised expression and secretion of DIII. **(A)** Scheme of constructs encoding DIII or DIII fused to the dimerising γCH3 domain. In both cases, sec indicates a signal leader peptide. The SV5 tag was included to facilitate detection. **(B)** Western blot (anti-SV5) of total cellular extracts (E) and supernatants (S) of HEK293T/17 cells transfected with the indicated constructs. **(C)** Western blot of supernatants (SN) of HEK293T/17 cells transfected with the same amounts of plasmid DNA, showing different secretion levels of the four DIII-CH3 proteins. **(D)** As in C, supernatants of HEK293T/17 cells transfected with DIII constructs with viral (V) or codon-optimised (CO) nucleotide sequences.

DIII from all four serotypes, initially derived from viral cDNAs (from amino acid 298 to 416 for DENV1 and DENV2, 296 to 415 for DENV3 and 298 to 416 for DENV4), were engineered adding a secretion leader peptide (sec) at the N-terminus (to allow translocation into the endoplasmic reticulum (ER) and the secretory pathway), and with or without the dimerising CH3 domain from the human IgG H-chain (γCH3) at the C-terminus (Fig 1A). To facilitate detection, the 11 aminoacid-long SV5-tag was also included. Expression and secretion of the proteins encoded in the different constructs were then tested in transiently transfected HEK 293 T cells. As shown in Fig 1B, DIIIs from the four serotypes were produced very poorly or not secreted and mostly retained intracellularly. In contrast, when fused to the γCH3 domain enhanced production and active secretion was obtained in all cases. We therefore adopted the DIII-CH3 format as the antigenic design to be used in plasmid-DNA immunisations. However, large differences in the efficiency of DIII-CH3 secretion were observed among the four serotypes (Fig 1C). With the exception of DIII from serotype 1 (1DIII), which was secreted at an acceptable level (1 μg/ml), all other serotypes showed much reduced secretion. In order to increase expression, DIII nucleotide sequences corresponding to serotypes 2, 3 and 4 were codon-optimised for expression in mammalian cells. Improved and comparable secretion levels for all serotypes were thus obtained (Fig 1D). As expected, improved antigen secretion resulted in a higher induction of antibody levels (S2 Fig).

### 2. Gene-gun immunisations induce DIII-specific antibody responses

**Fig 2:**
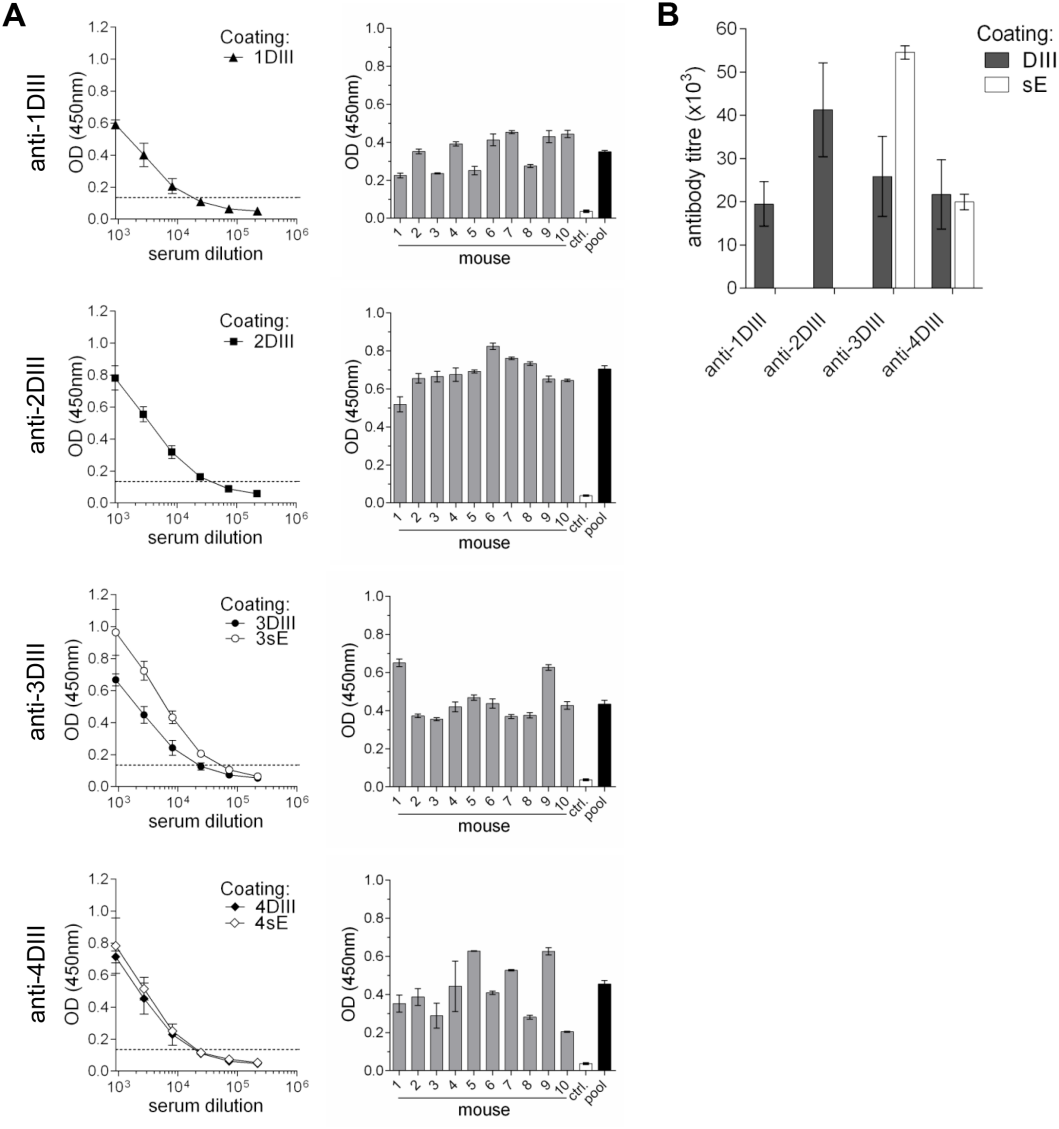
Antibody responses by conformational ELISA. **(A)** ELISA reactivity of pooled sera performed on plates coated with the distinct DIII serotypes (left panels) and individual sera of gene-gun immunised mice (OD_450_ at a 1:2700 dilution) compared to the pool (right panels). Ctrl.: negative control sera. Reactivity against 3sE and 4sE proteins, coated at the same molar concentration as DIII, is also shown in anti-3DIII and anti-4DIII left panels. **(B)** Plot of titres of the four different serotypes pools, determined on DIII and sE (for serotypes 3 and 4).

Groups of 10 animals for each serotype were immunised by intradermal gene-gun delivery of the DIII-CH3 plasmid DNA construct, with a total of three shots with 1μg of DNA each, at 15 days intervals. Mouse sera were then tested for anti-DIII antibodies in a conformational ELISA (Fig 2A) using *in vivo* mono-biotinylated recombinant proteins secreted from mammalian cells, containing either DIII (for all four serotypes) or the full soluble E ectodomain (sE, E sequence without the C-terminal amino acids from the last half of the stem and anchor regions), of the best secreted serotypes 3 and 4 (3sE and 4sE, respectively). DIII were fused to the CH4 domain of human IgE H-chain (εCH4), which also supports secretion, but is not cross-reactive with anti-γCH3 antibodies. Both, DIII-εCH4 and sE proteins were fused at the C-terminus to the biotin acceptor peptide (BAP [32]). sE molecules contained at the C-terminus also a tag for detection (roTag [35]) (S3(A) Fig). These proteins were expressed and secreted by stably transfected clones (in HEK293 cells) co-expressing the biotin ligase sec-BirA, which catalyses the covalent ligation of biotin into the single Lys residue within BAP, as previously reported [33]. The biotinylated proteins were captured on avidin-coated plates (schematically shown in S3(B) Fig). Sera were tested in plates containing comparable amounts of recombinant DIII.

**Fig 3:**
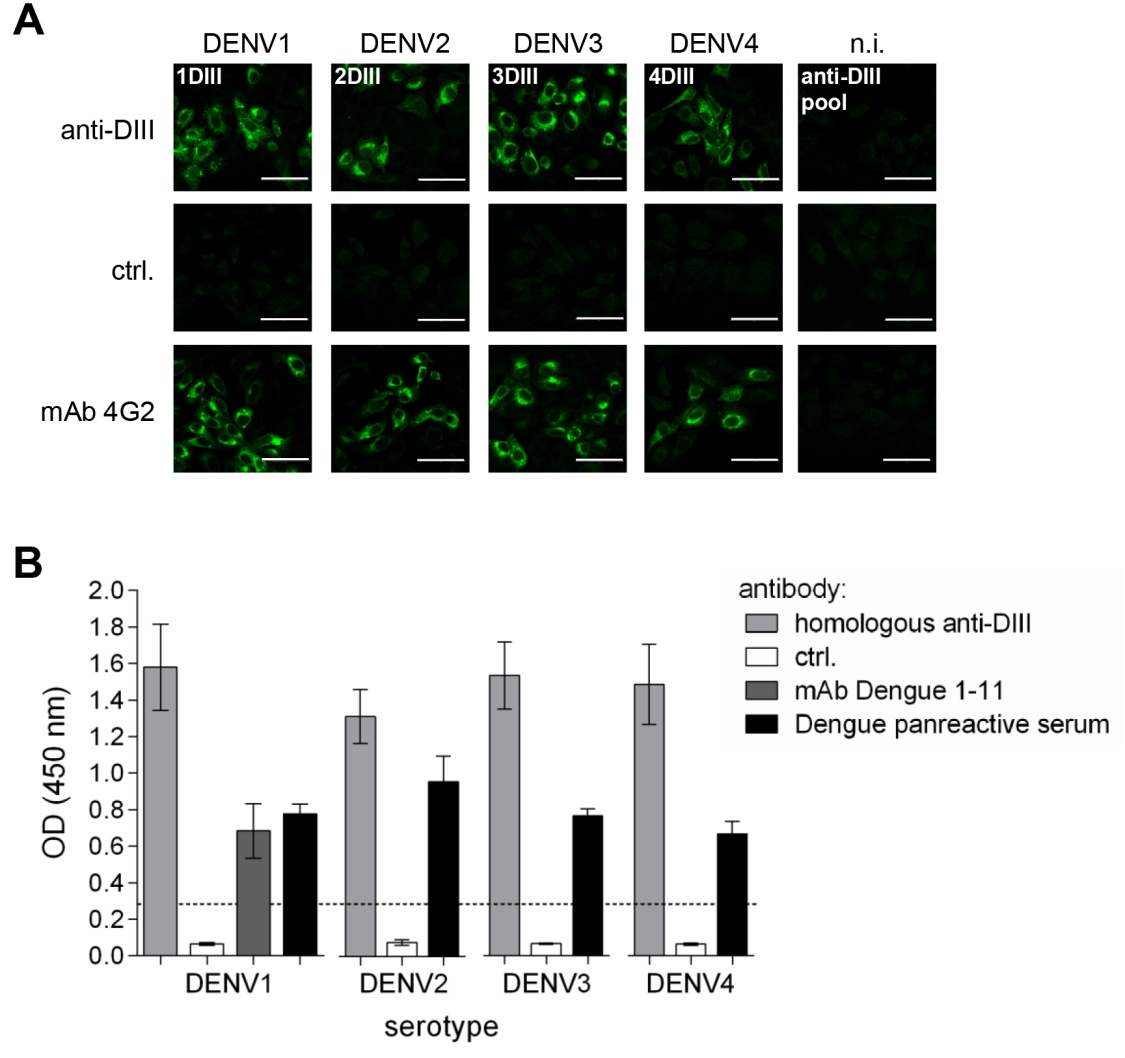
Anti-DIII sera recognise viral E protein. **(A)** Immunofluorescence of Vero cells infected with DENV of each serotype, reacted with the serotype-specific anti-DIII pools of sera (top row), the negative control sera (ctrl., middle row) and mAb 4G2 (bottom row). In each case, non-infected cells (n.i., rightmost column) were also used as controls. Bars represent 50 μm. **(B)** ELISA on whole infective viral particles. Anti-DIII serotype-specific pools diluted to 100 ng/ml and sera from mock-immunised animals were used onto virus particles captured on plates coated with a human serum reactive against all four serotypes. mAb Dengue 1–11 reactive against DENV1 E (at 1 μg/ml) and a Dengue pan-reactive serum against all four serotypes were used as positive controls.

Anti-DIII antibodies from all serotype groups were elicited at high titres (expressed as the dilution producing an optical density at 450nm (OD_450_) threefold higher than the negative control serum), with values ranging from 1:19500 (for anti-1DIII) to 1:41300 (for anti-2DIII) (Fig 2B), corresponding to a range of antigen-specific antibody concentrations between 16–35 μg/ml, respectively (Table 1). This was true for sera collected at both time points (days 45 and 60). These antibodies were also able to recognise DIII in the full-length sE protein in ELISA (shown in Fig 2A for 3sE and 4sE). In addition, immunofluorescence microscopy revealed that all four different serotype-specific sera recognised E protein in virus-infected Vero cells (Fig 3A). Further confirmation of reactivity with protein E was obtained by ELISA on infective viral particles that were captured on plates coated with a serum reacting against the four different viral serotypes. All sera were used at an antibody concentration of 100 ng/ml (Fig 3B). These results indicate that a substantial antibody response was directed against DIII epitopes exposed in the complete envelope ectodomain. The different pools of sera were also able to bind to DIII domains displayed on the cell surface membrane through a glycosyl-phosphatidylinositol (GPI) anchor in Sp2/0 stably transfected clones, detected by cytofluorimetry (S4 Fig).

**Table 1:** Anti-DIII specific concentration in sera from vaccinated mice. Anti-DIII ELISA titres expressed as antibody concentrations, obtained from dilution curves compared to mAb 4G2.

Immunogen	Coating	[Antibody] (mg/ml)
1DIII-CH3	1DIII	(1,6 ± 0,3) x 10^−2^
2DIII-CH3	2DIII	(3,5 ± 0,9) x 10^−2^
3DIII-CH3	3DIII	(2,1 ± 0,3) x 10^−2^
4DIII-CH3	4DIII	(2,0 ± 0,1) x 10^−2^

**Fig 4:**
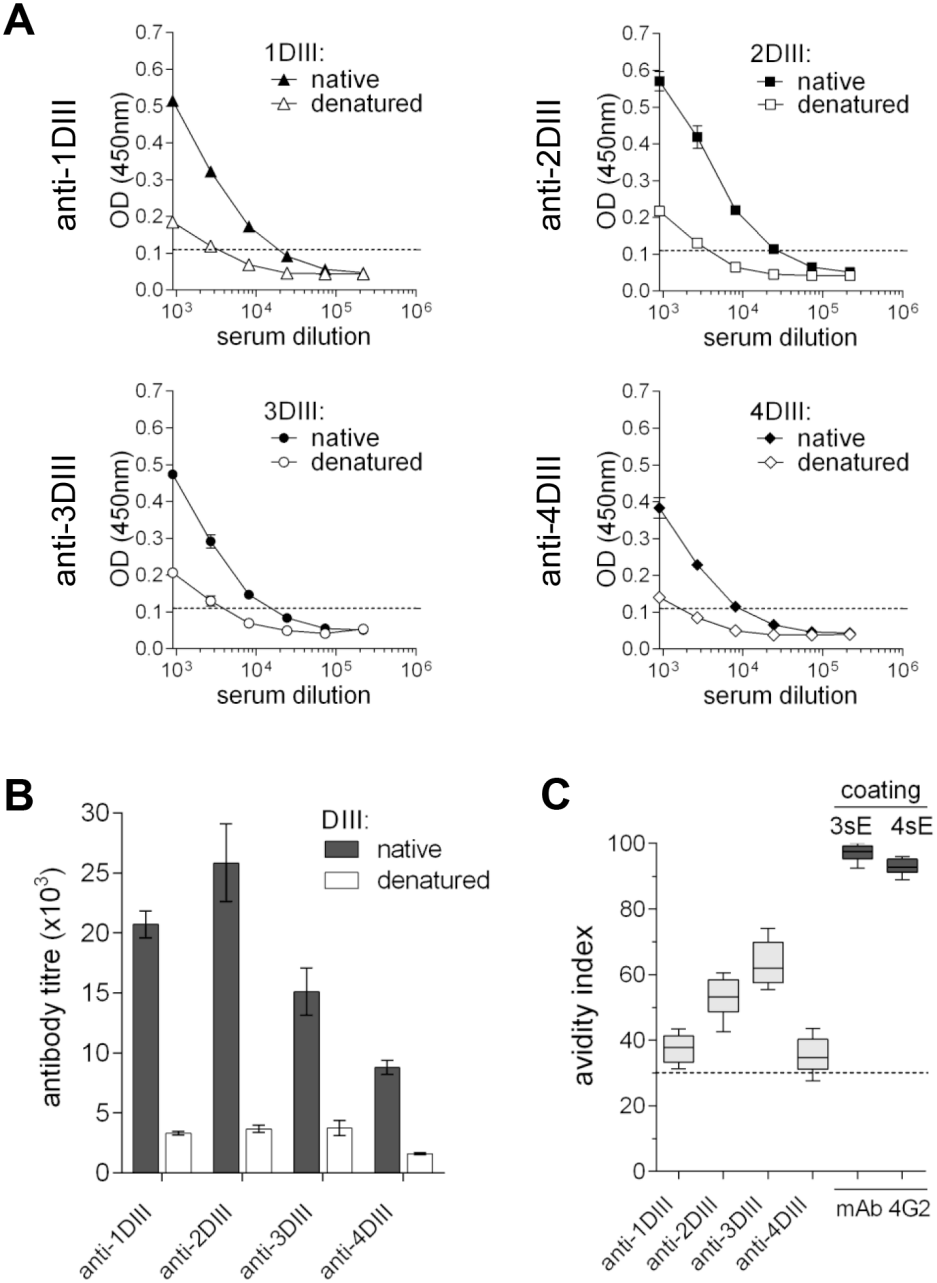
Anti-DIII sera recognise conformational epitopes with high avidity. **(A)** Equal amounts of native or denatured biotinylated DIII-εCH4 were captured on avidin-coated plates and reacted with the corresponding homologous anti-DIII sera. **(B)** Plot of the titres from the curves shown in A. **(C)** Avidity index of each anti-DIII sera determined on the native homologous DIII-εCH4. Avidity index of mAb 4G2 on 3sE and 4sE is shown as a control.

These results suggested a conformational nature of the induced antibodies. In fact, all four sera lost most of their reactivity towards the denatured antigen, as compared to the native one, when tested by ELISA with the same amount of coating proteins (Fig 4A). The comparative antibody titres determined on native and denatured antigens are shown in Fig 4B. The avidity index of each serum was also determined performing ELISA in stringent dissociating conditions with 6M urea. All four sera showed an avidity index close or well above 30, indicating the presence of a substantial concentration of high affinity antibodies [41] (Fig 4C). The avidity index of the control anti-dengue mAb 4G2 was also determined on 3sE and 4sE proteins which resulted in indexes close to 100 in both cases.

### 3. Cross-reactivity of serotype-specific sera

**Fig 5:**
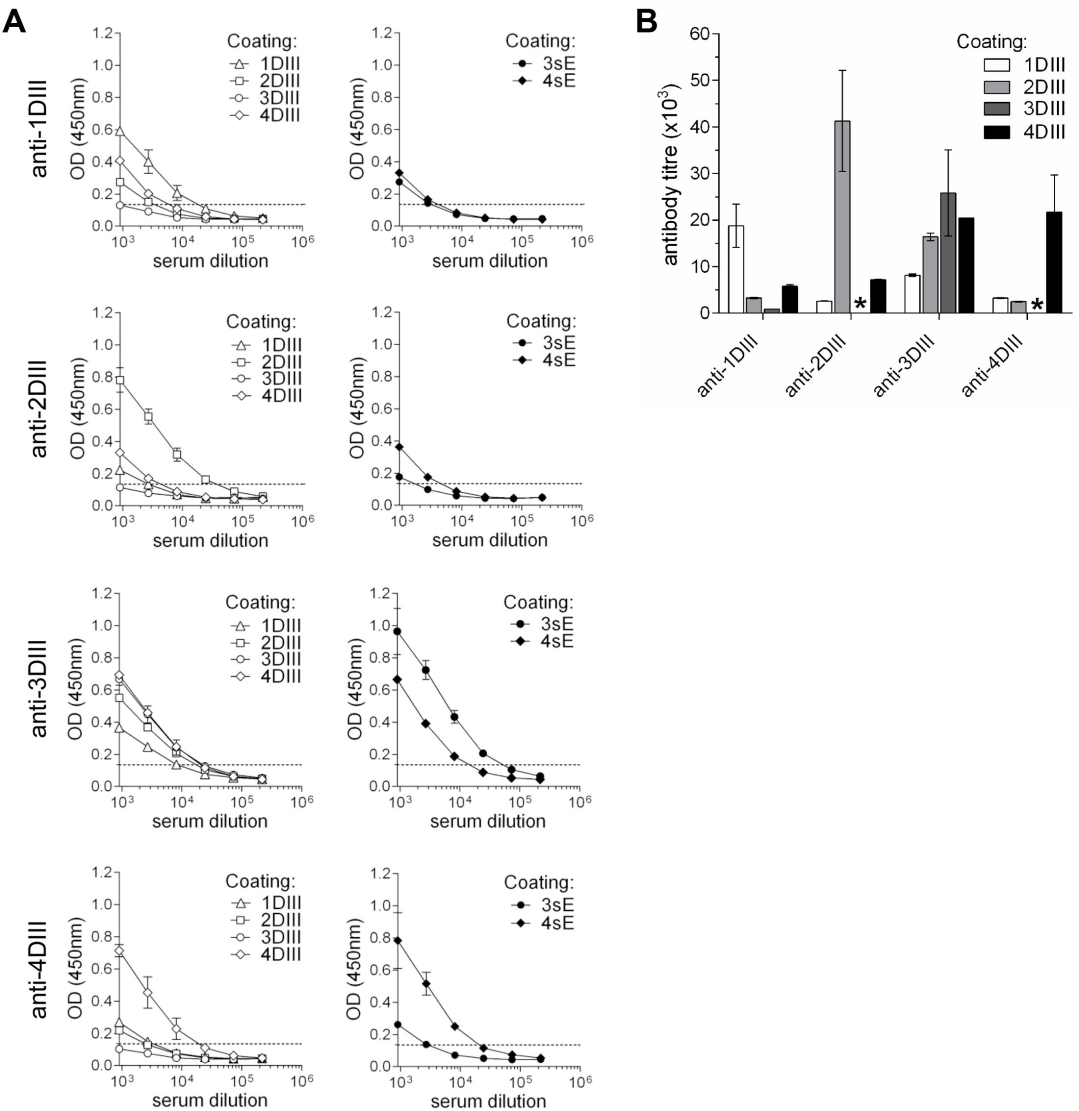
Cross-reactivity of anti-DIII sera. **(A)** ELISA reactivity of the different serotype specific anti-DIII sera on the four DIII-εCH4 (left panels) and on the two secreted sE (3sE and 4sE), (right panels). **(B)** Cross-reactive titres from the curves shown in A. * indicates titre below control (absence of cross-reacting antibodies).

In order to determine the capacity of each serotype in inducing cross-reactive antibody responses against other serotypes, sera from each group were tested against all serotypes by conformational ELISA on DIII or the 3sE and 4sE ectodomains. As shown in Fig 5A, each pool of serotype-specific anti-DIII showed some degree of cross-reactivity against the different serotypes. As expected, all sera showed the highest reactivity against the homologous antigen. Yet, the cross-reactivity profiles were not the same. While serotype 1 antibodies were mostly cross-reactive with serotype 4 DIII, they reacted much less with serotypes 2 and 3. Serotype 2 antibodies instead, showed lower cross-reactivity (the most significant with 4DIII). A similar low cross-reactivity was observed for serotype 4 sera. In contrast, serotype 3 antibodies were highly cross-reactive with 4DIII and 2DIII and somehow less with 1DIII. The antibody titres of each pool of serotype-specific sera determined on all four different DIII are plotted in Fig 5B. The data in Table 2 summarises the reactivity of each serum relative to the value of the homologous serum. For instance, anti-3DIII serum showed a titre against 1DIII of around 43% of the corresponding homologous serum, while it showed a higher value for 4DIII (94%), suggesting that immunisation with 3DIII could significantly contribute to increase the anti-4DIII response.

**Table 2:** Relative cross-reactivity of serotype-specific anti-DIII sera. Reactivity against each serotype DIII-εCH4 antigen expressed in relation to the reactivity against the homologous antigen, taken as 100%.

Coating	Serum			
	anti-1DIII	anti-2DIII	anti-3DIII	anti-4DIII
1DIII	100	14	43	17
2DIII	8	100	40	6
3DIII	3	ND*	100	ND*
4DIII	27	33	94	100

ND*: ELISA reactivity not detected

### 4. Anti-DIII antibodies have virus neutralising activity

**Fig 6:**
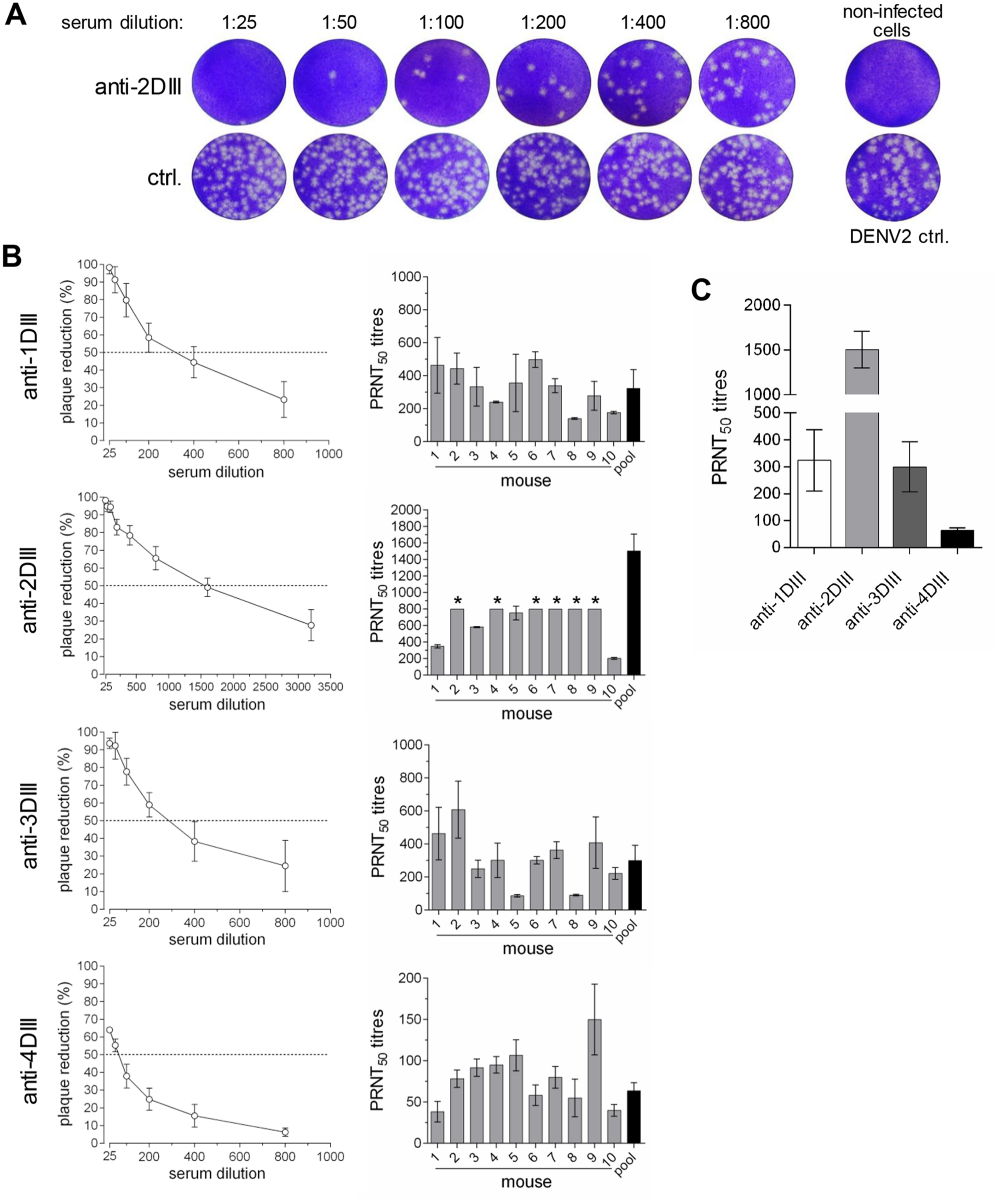
Virus-neutralising activity of anti-DIII sera. **(A)** Plaque reduction neutralisation test (PRNT) of DENV2 on Vero cells with sera from mice immunised with 2DIII-CH3 or mock-immunised. **(B)** Plaque reduction curves (left panels) using pools of sera from the different groups of animals, relative to negative control sera with PRNT_50_ titres of each animal serum (right panels) (* indicates PRNT_50_ titre is higher than 800). **(C)** PRNT_50_ titres from curves shown in B.

Neutralising activity of the serotype specific sera was tested implementing the plaque reduction neutralisation test (PRNT) for each virus serotype in Vero cells. Fig 6A shows a representative set of plates with plaques for DENV2, not treated or treated with mouse anti-2DIII serum or a mouse negative control serum. Neutralisation titres (taken as fold dilution producing 50% reduction of plaques, PRNT_50_) of each group of animal sera were first determined against the homologous DENV serotype.

All animals within each group showed neutralisation titres (Fig 6B right panels), albeit with some differences. The neutralisation curves for the four different pools (Fig 6B, left panels) indicated high titres for serotypes 1 (300), 2 (1600) and 3 (300) and a lower value for serotype 4 (65). Antibody titres are summarised in Fig 6C. Thus, all four DIII-CH3 constructs were efficient in inducing neutralising responses against the homologous DENV serotype. This high efficiency was mostly due to the level of secretion of the DIII domain, as constructs that were not codon-optimised, and therefore produced and secreted at much lower levels, induced lower immune responses (S2 Fig).

### 5. Secretion and immune response

**Fig 7:**
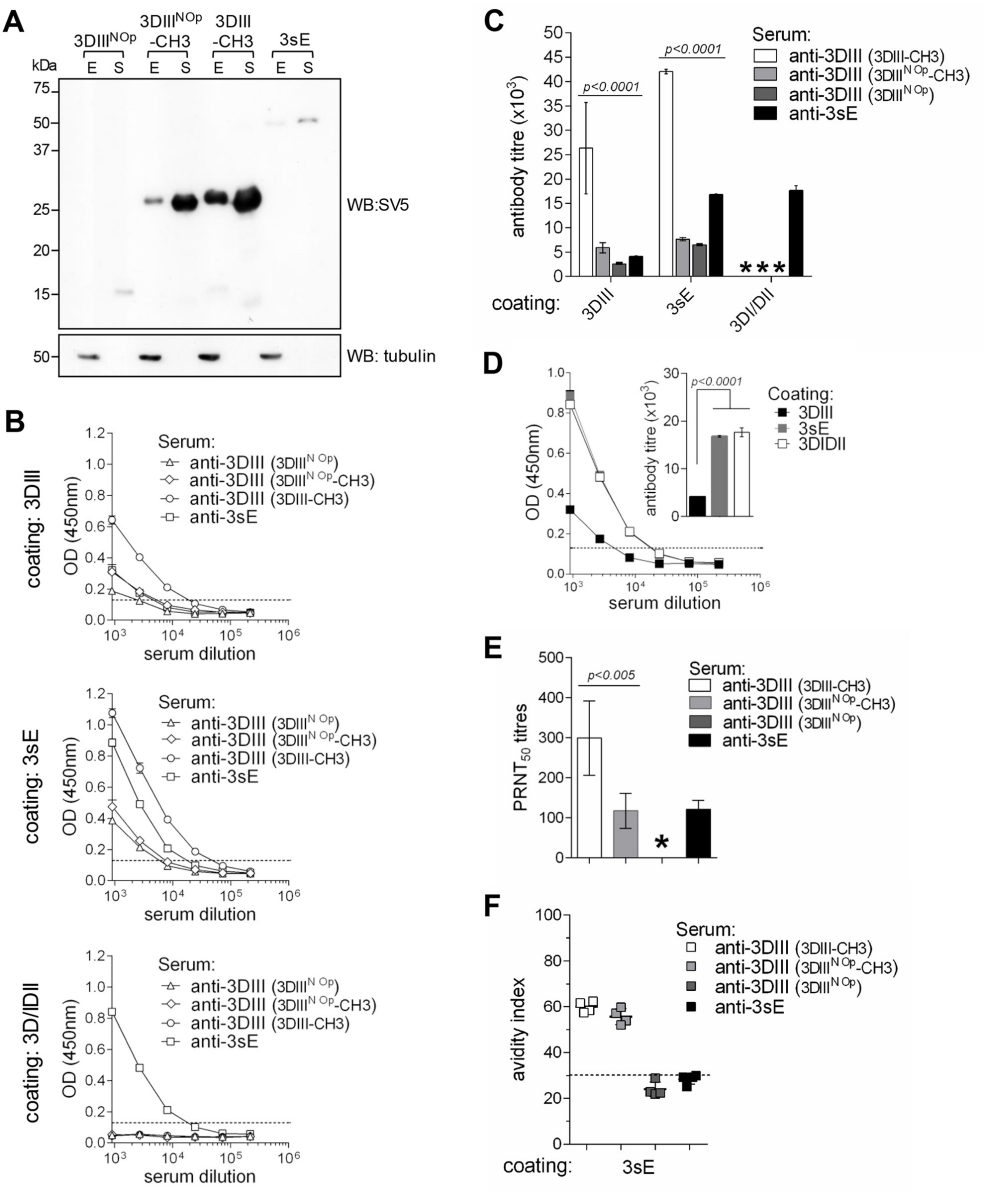
Comparison of antibody responses of 3DIII and 3sE antigens. **(A)** Western blot of total cellular extracts (E) and supernatants (S) of HEK293T/17 cells transfected with plasmid constructs encoding 3DIII^NOp^ (~16 kDa), 3DIII^NOp^-CH3 (~28 kDa), 3DIII-CH3 (~28 kDa) and 3sE (~54 kDa). **(B)** ELISA of sera derived from mice gene-gun immunised with 3DIII^NOp^, 3DIII^NOp^-CH3, 3DIII-CH3 or 3sE (immunising antigens indicated in parenthesis) tested on plates coated with biotinylated versions of 3DIII-εCH4 (3DIII), 3sE and 3DI/DII. **(C)** Antibody titres determined on each of the different coating proteins, from the curves shown in B (* indicates no reactivity detected). **(D)** Plot of anti-3sE sera reactivity (from 3sE immunised animals) on the three different coating proteins: 3DIII, 3sE and 3DI/DII. Insert: anti-3sE titres for each coating protein. **(E)** PRNT_50_ titres of sera from mice immunised with 3DIII^NOp^, 3DIII^NOp^-CH3, 3DIII-CH3 or 3sE (immunising antigens indicated in parenthesis) tested on DENV3. **(F)** Avidity index of antibodies derived from animals gene-gun immunised with 3DIII^NOp^, 3DIII^NOp^-CH3, 3DIII-CH3 or 3sE (immunising antigens indicated in parenthesis) tested on 3sE-coated plates.

We then set out to compare the immune responses elicited by four different constructs encoding DIII from serotype 3 in distinct contexts: the preferred codon-optimised 3DIII-CH3, the same one with non codon-optimised DIII (3DIII^NOp^-CH3), the non codon-optimised DIII alone (3DIII^NOp^) and the 3sE. The secretory phenotype, tested in transfected HEK293T/17 cells, showed that 3DIII-CH3 was clearly the one secreted at highest levels (Fig 7A). Sera from groups of 5 animals vaccinated with each construct were tested by ELISA, determining anti-DIII antibodies (plates coated with biotinylated 3DIII-εCH4), anti-E antibodies (plates coated with biotinylated 3sE) and anti-DI/DII antibodies (plates coated with 3DI/DII, an sE version with DIII deleted). As shown in Fig 7B, the responses of animals vaccinated with DIII-CH3 were higher than those obtained with DIII^NOp^-CH3, DIII^Nop^ alone or with sE when tested on DIII (topmost panel) and on the sE protein (middle panel). As expected, only sE induced antibodies strongly reacting with DI/DII protein (bottommost panel). Antibody titres are summarised in Fig 7C. As shown in Fig 7D the reactivity of the anti-sE antibodies towards DI/DII was higher than to DIII and comparable to sE (Fig 7D, insert). Conversely, despite its focused reactivity towards DIII, the neutralisation titre (on DENV3) of sera induced with DIII-CH3 was significantly higher than the one obtained with sE, further highlighting the crucial role of DIII as a target for neutralising responses (Fig 7E). Moreover, the avidity indexes of anti-DIII antibodies induced with DIII-CH3 and DIII^NOp^-CH3 were significantly higher (60) than the one induced with DIII^NOp^ alone, thus indicating the importance of the CH3 domain (Fig 7F) (24). To normalize conditions, these assays were all performed with equal amounts of coated proteins.

### 6. Cross-neutralisation and ADE activity of serotype-specific sera

In order to investigate cross-neutralisation activity towards the non-homologous serotypes, each pool of sera was also tested against the other DENV serotypes using the classical PRNT_50_ in Vero cells. The results obtained are summarised in Table 3. Similarly to the data obtained on the ELISA cross-reactivity, serotype 1, serotype 2 and serotype 4 antibodies did not show significant cross-neutralising activity (<10 for serotypes 2 and 4 and <25 for serotype 1 against all others), while serotype 3 antibodies did show significant neutralisation of DENV4 (titre ≈30) and DENV2 (titre ≈135) but not to DENV1 (titre <25) despite being cross-reactive.

**Table 3:** Anti-DIII cross-neutralising activity. PRNT_50_ titres of each serotype-specific anti-DIII pool determined on all four serotypes.

Serum	Viral Serotype			
	DENV1	DENV2	DENV3	DENV4
anti-1DIII	≈300	<25	<25	<25
anti-2DIII	<10	≈1600	<10	<10
anti-3DIII	<25	≈135	≈300	≈30
anti-4DIII	<10	<10	<10	≈65

**Fig 8:**
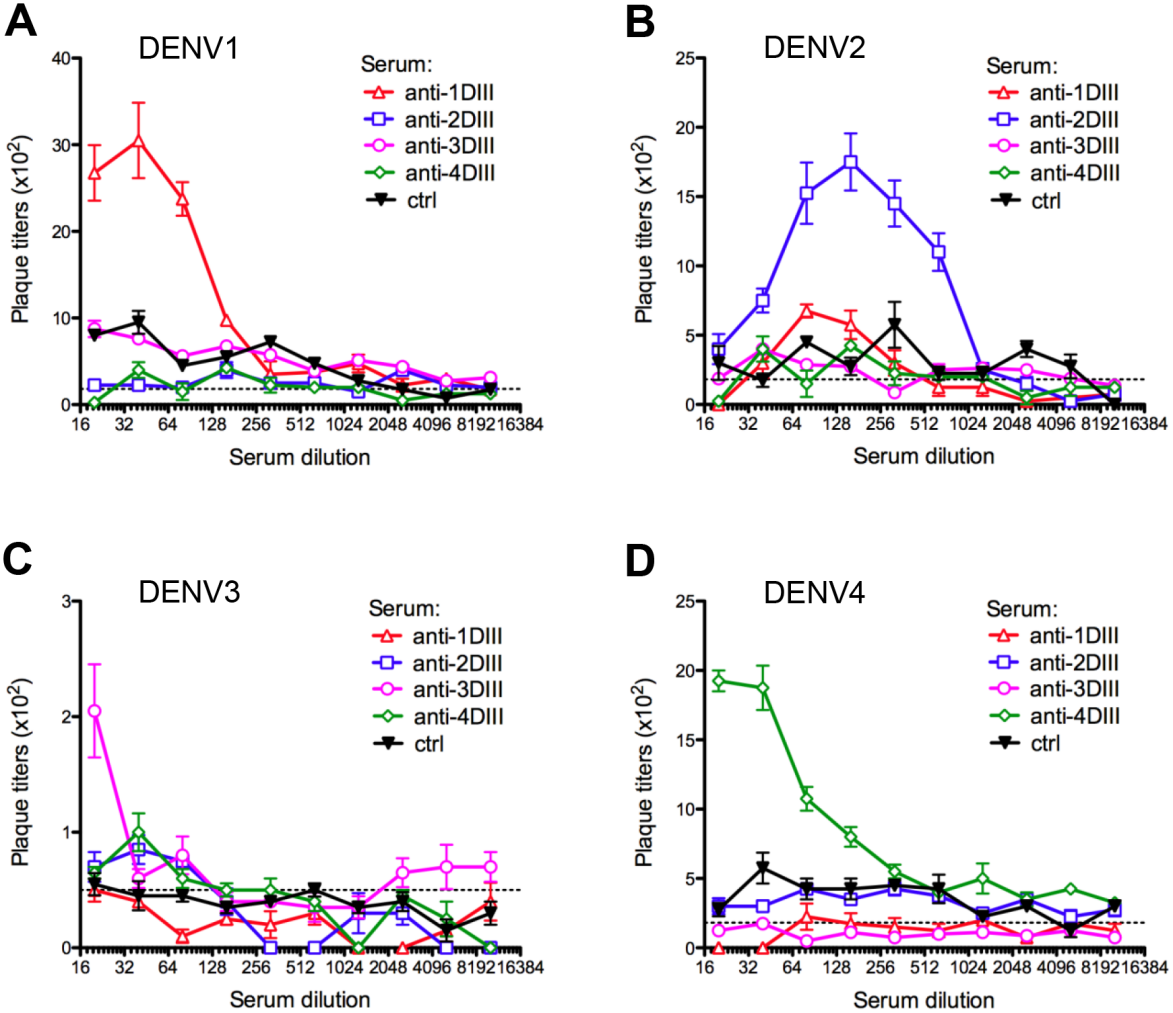
Comparison of antibody-dependent enhancement responses of sera in monocytes. **(A-D)** Each serotype-specific anti-DIII pool and control sera were diluted two-fold and incubated with **(A)** DENV1, **(B)** DENV2, **(C)** DENV3 and **(D)** DENV4 before infecting THP-1 cells for 72h. After which, the culture supernatant was quantified for DENV using plaque assay. Dashed line indicates DENV infection alone of THP-1 cells without the addition of any serum sample.

To further assess the functional specificity of the antibody responses and to determine if the antibody response to our vaccine would cross react with and enhance infection of heterologous DENV serotypes, we also performed an antibody-dependent infection assay. Each serum sample was serially diluted and incubated with each of the four DENV serotypes before inoculating each reaction onto the human monocytic cell line THP-1 that expresses FcγRI and FcγRII [9]. Enhancement of DENV titres was observed only to the homologous but not to the heterologous serotypes of DENV (Fig 8), indicating functional specificity in antibody binding. As expected, the control serum samples did not enhance DENV titres in THP-1 cells.

### 7. Neutralising antibodies of tetravalent formulation

**Fig 9:**
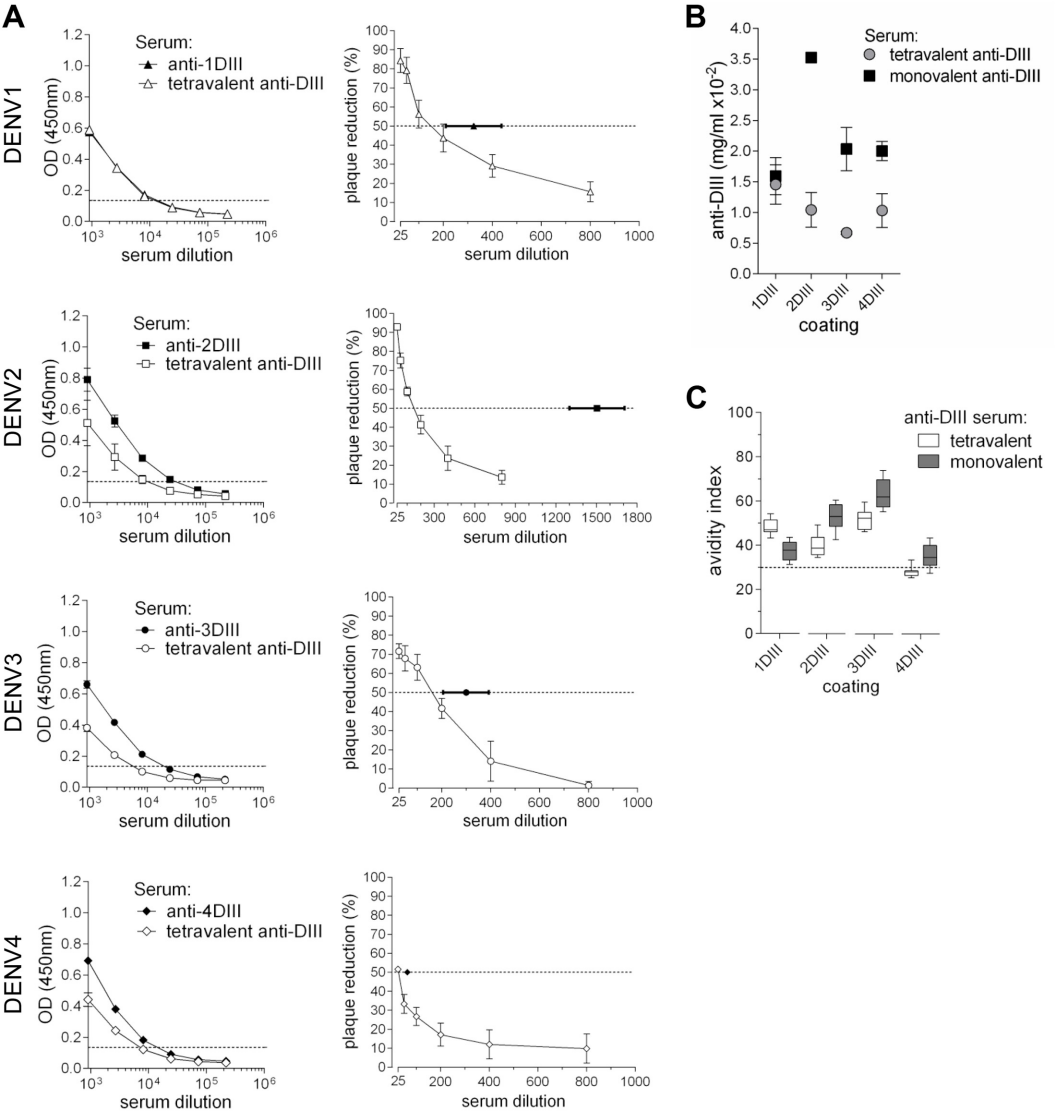
Tetravalent formulation. **(A)** ELISA (left panels) and PRNT_50_ (right panels) of pools of sera from animals gene-gun immunised with a single DIII-CH3 construct or with the tetravalent formulation. Filled and open symbols indicate monovalent and tetravalent immunisations, respectively. In right panels, curves correspond to the tetravalent vaccine and the PRNT_50_ titres from the monovalent immunisations (determined in Fig 6) are shown for comparison. **(B)** ELISA titres (expressed as anti-DIII antibody concentrations) from monovalent and tetravalent immunisations, determined on each serotype. **(C)** Avidity index of sera from monovalent and tetravalent immunisations, determined on the different DIII serotype antigens.

Based on the responses obtained with each of the different immunogens, we then tested a tetravalent formulation. The tetravalent vaccine contained a mix of the four serotype-specific genetic constructs. Using the same vaccination protocol, the total amount of DNA per dose was increased to 2 μg (two shots of 1 μg per shot, containing a mix of serotypes 1 and 2 and a mix of serotypes 3 and 4, respectively); thus each construct was present at 50% of what delivered alone (0.5 μg). After three immunisations, a pool of sera from 5 vaccinated animals was tested by ELISA and PRNT. As for the individual constructs, the tetravalent vaccine was able to induce DIII-specific antibody titres (Fig 9A left panels) with high avidity indexes and a balanced neutralising activity (PRNT) against all four serotypes (Fig 9A right panels, and Fig 9C). Despite the reduction in antibody concentration and neutralisation titres in comparison with monovalent vaccines (Fig 9A right panels and Fig 9B), this is a first attempt of a tetravalent formulation and represents a proof of principle, with potential for further optimisation.

## Discussion

In this paper we show that immunisation with DNA plasmid constructs encoding properly engineered DIII domains of the four DENV serotypes can induce strong antibody responses in mice. In the context of the immune response against DENV E protein, the Ig-like DIII has been shown to be one of the main targets for protective neutralising antibodies. Highly neutralising epitopes have also been found in regions involving DI, DII [24, 44, 45] and, more recently, dimer-dependent epitopes at the interface between the opposing E monomers [46, 47] were described. However, antibodies reactive with DI/DII were shown to be more cross-reactive, with lower neutralisation potency and consequently implicated in enhanced severity of infection [39, 48]. Recent studies have shown that in virus infected individuals the antibody response is dominated by highly cross-reactive antibodies, while antibodies directed against the more specific DIII represent only a minor component [24]. Because of the increased risk of ADE due to the presence of such cross-reactive antibodies, several attempts for an efficient anti-dengue vaccine have focused on the use of the highly specific DIII as antigen [49].

In designing our DIII DNA-based vaccine we took into consideration the biochemical properties of the antigen, as it has to be expressed in and secreted from the cells of the host. A good level of protein expression and secretion is required to induce a strong immune response via DNA vaccination [25, 26, 43]. A leader signal peptide was fused to the DIII N-terminus to direct its translocation to the ER lumen and the secretory pathway. However, robust secretion was only achieved when DIIIs were fused at the C-terminus to the dimerising γCH3 and further increased upon codon optimisation for expression in mammalian cells of the encoded DIIIs. The CH3 domain played a crucial role in allowing intracellular transport of the recombinant proteins leading to their efficient secretion. This was essential for serotypes 2 and 4, which were otherwise essentially not secreted, and very important for serotypes 1 and 3, whose secretion was strongly increased. Interestingly, by fusing to CH3 the quality of the immune response (in terms of avidity index) was also improved.

We immunised mice with plasmid DNA delivered through gene-gun technology. Our results confirm that this is a very efficient way to induce, with very low amounts of total DNA, highly specific antibody responses, which are mainly directed against conformational epitopes exposed on the infective viral particle. This was indeed shown in experiments where either the virion or the same amounts of native and denatured coated antigen were used to determine sera reactivity. This was also reflected, in part, in the relatively low cross-reactivity of each serotype specific serum. We took special care in the design of the ELISA to test antibody responses. The proteins used were all exclusively produced and secreted from mammalian cells in a mono-biotinylated form that did not require any further purification. Dialysed culture supernatants were used as the source of proteins that were then captured on plates coated with avidin. Thus, antibodies detected in this assay corresponded to those reacting mainly with conformational epitopes on the folded antigen. In fact, the anti-DIII sera elicited with each serotype were able to react also with the whole sE ectodomain (shown for the DENV3 and DENV4) expressed in mammalian cells and with the whole infective viral particle (for all four serotypes).

Given the impossibility of comparing ELISA data across dengue vaccine-related studies, we decided to translate our ELISA reactivity data into estimated antibody concentrations, in an attempt to promote the use of measurements and methodologies that allow the establishment of parallelisms between different vaccine candidates. All four serotypes induced high antibody concentrations; in particular serotype 2 was the one producing the highest responses, both in antibody concentrations as well as in neutralisation titres. This was in part due to the contribution of the optimised secretion levels, which otherwise induce very low titres, as well as the site of immunisation. Gene-gun technology delivers DNA intradermally, transfecting mainly keratinocytes that produce and secrete the antigen in an immunologically favourable environment [50]. Availability of antigen, as reflected by the secretion levels, was important. In fact, optimised DIII-CH3 elicited stronger responses than the one with non-codon optimised DIII, which was in turn stronger than the DIII alone. This was also reflected in the neutralisation titres. Additionally, the dimeric structure of the immunogen as a result of its fusion to the γCH3 domain, could also be in part relevant, as it would favour engagement of the B-cell receptor (BCR) and subsequent activation of naive B cells. In addition, the xenogeneic nature of the CH3 domain contributes to an increased activation of T helper cells [51]. Thus, when analysing the contribution of the modifications introduced during the design of the antigen, we confirmed that codon optimisation improved antibody titres by increasing antigen secretion, while fusion to CH3 improved immunogenicity and secretion levels of the antigen as well as the avidity index and neutralising capacity of the induced antibodies.

It has been shown that neutralisation of virions in flaviviruses follows a “multi-hit” requirement model, in which the number of bound antibodies must surpass a required threshold [10, 12]. This threshold is different for each epitope and is mainly determined by the combination of two biochemical factors: antibody avidity and accessibility of the epitope on the virus [52]. Antibody avidity and *in vitro* neutralising activity were shown to positively correlate for anti-Flavivirus monoclonal antibodies [10, 53–55], and for antibodies to other viral infections [56, 57]. Recently, this correlation was also demonstrated in sera of DENV-infected patients [58]. Our results confirm these observations as the DIII-CH3 DNA immunisations induced polyvalent antibody responses in which the neutralising capacity (determined as PRNT_50_ titres) correlates better with the respective avidity indexes than with the sera reactivity measured in ELISA. These data thus support not only the use of specific avidity indexes, but also the idea of introducing these measurements into the evaluation of vaccine candidates, especially for DENV [58].

Several DIII-based dengue vaccines have been reported, using different vaccination strategies including recombinant protein subunit vaccines [59–68], DNA vaccines [69–71] or viral-vectored live vaccines [72, 73]. In our case, we have emphasised the design and evaluation of the antigen’s biochemical properties necessary to improve immunogenicity. As shown here, our DIII-CH3 DNA vaccine was able to induce stronger neutralising responses against all four serotypes as compared to other DIII-based vaccines [60, 69], even those based on protein immunisation without or with DNA boosting. In addition, the cross-neutralisation profile obtained in the PRNT_50_ in Vero cells was similar to the cross-reactivity profile obtained by ELISA, suggesting that most of the cross-reacting antibodies were also cross-neutralising. This is in agreement with recent data showing that cross-reactive antibodies contribute to neutralisation during acute DENV infections [58]. In our case, serotype 3 anti-DIII antibodies were the most cross-reactive and showed the highest cross-neutralisation towards the other serotypes. Noteworthy, the ADE assay revealed that each of the DIII-specific sera were not able to enhance infection of heterologous DENV serotypes in the monocytic cell line THP-1, demonstrating serotype specificity in our DIII-CH3 DNA vaccine. Without antibodies that enhance heterologous DENV serotype infection, ADE would hence only occur when homologous antibodies decay to sub-neutralising levels. For clinical administration of the tetravalent formulation, further optimisation should be performed to ensure that the antibodies are produced at levels that do not result in ADE.

Various studies have proposed that genotype differences within each serotype could affect vaccine efficacy [54, 55, 74–76]. In a recent phase 2b study conducted in Thailand, failed protection against DENV2 (9.2%) was hypothesised to be due to differences in the circulating genotype [77], while in a more recent phase 3 study conducted in Latin America vaccine efficacy for DENV2 was reported to be higher [78]. Interestingly, the neutralisation titres we obtained for DENV2 were the highest among all four serotypes in the monovalent immunisations and remained high in the tetravalent one. If genotype differences represent an important issue to obtain wide protection against defined serotypes, DNA vaccines are particularly adapted to easily introduce appropriate changes, what represents a significant advantage when compared to other vaccination strategies.

Despite the fact that antibodies against DIII have greater neutralising capacity, most of the vaccine candidates for DENV use the whole E protein ectodomain (with or without PrM) as an antigen [79]. In this regard, recent data indicate that the antibody response against dengue is dominated by highly cross-reactive antibodies that are mainly focused on antigenic determinants around DII [45, 48]. Specifically, the main neutralising targets in the response against the E protein, involve epitopes located in or around the fusion loop and the DI/DII hinge region [39, 44, 48, 80]. Considering this, we compared the immune response elicited by our DIII-CH3 construct with that of the whole E ectodomain. Our data proved that the DIII-CH3 construct was able to induce a stronger antibody response and also confirmed that, when using the E ectodomain as antigen, the antibody response was shifted towards DI/DII with a significantly weaker response against DIII. As a consequence, neutralising responses induced by our DIII-CH3 were significantly higher than the ones elicited in animals vaccinated with the E ectodomain (sE). In fact, when compared to other DNA vaccines against dengue that use the complete E protein as their main antigen [81–85], our DIII-based vaccine, despite delivering significantly lower amounts of plasmid DNA per immunisation, was able to elicit higher neutralising immune responses in mice. Moreover, the DIII-CH3 candidate still shows higher efficiency when compared to other DNA vaccines where the E protein was further modified to enhance immunogenicity [86–91]. The only exception was the DENV4 neutralising response, which was lower than the ones reported by others [82–84, 88].

As a proof of principle we attempted to formulate a tetravalent immunisation protocol. The results showed an efficient anti-DENV activity elicited against all four virus serotypes, despite the reduced amount of DNA used for each serotype. As with the monovalent formulation, the lowest neutralising activity in the tetravalent formulation was against DENV4. This appears to be a characteristic of the whole 4DIII domain, as immunisations with 4DIII variants derived from two different genotypes (strain Dominica from genotype II and strain TC25 from genotype I) with three amino acid differences (L357F, N360Y and N384D) produced similar results and comparable neutralisation titres when tested on strain TC25 (S5 Fig). This indicates that further development of the tetravalent formulation is needed to reach comparable levels of neutralisation activities against all four serotypes. Some aspects to consider include the total amount of DNA, the relative proportion of each plasmid and the sites of administration.

In conclusion, we think that at least four key points in our protocol contributed to the high responses observed. i) First, the genetic nature (DNA) of the immunisation, which is capable of inducing long-term humoral and cellular immune responses by effectively surrogating the viral infection process. ii) Second, engineering of the antigen molecule to make it available to the immune system in significant amounts, since our DIII-CH3 codon-optimised version is actually secreted at much higher levels as a dimeric molecule. iii) Third, the use of DIII as immunogen instead of the whole E ectodomain, thus reducing the level of cross-reactive non-neutralising antibodies and consequently, the risk of ADE; and iv) Fourth, the intradermal delivery of the plasmid DNA that elicits a balanced Th1/Th2 response, as opposed to intramuscular delivery which mainly activates the Th1 pathway [92].

As live vaccines candidates against dengue remain disappointing in clinical trials, next-generation vaccines have emerged as new alternatives with the potential to succeed where the classical strategies have failed. Since the first clinical trial for a DNA vaccine against HIV-1 virus in 1998 [93], several other DNA vaccine candidates developed against infectious diseases have been tested in Phase 1 studies [94–98]. To date there has been only one published dengue DNA vaccine clinical trial involving a Phase 1 study of a plasmid expressing the PrM and E proteins of DENV1 (D1ME100, [99]). In all cases, the studies showed that the vaccines were well-tolerated and safe in humans [100], although low immunogenicity remains a main concern associated with DNA vaccines [100, 101].

Compared to other vaccination strategies, genetic vaccines are safer, more stable, easier to manipulate and have a relatively low production cost. These represent important aspects to consider when designing vaccines for developing countries [100, 102]. We believe that accurate design of the antigen and the ability to induce the right antibody response avoiding the undesirable non-neutralising cross-reactive ones are key points to develop for a successful DNA vaccine.

## Supporting Information

S1 Fig Amino acid sequences for the four different DIII DENV serotypes. Amino acids conserved across the four serotypes are highlighted.
(TIF)

S2 Fig Effect of codon-optimization on the immune response elicited by DIII-CH3 antigens. **(A-C)** ELISA of sera from animals gene-gun immunised with DIII-CH3 constructs from serotypes 2 **(A)**, 3 **(B)** and 4 **(C)**, with viral (DIII^NOp^-CH3) or codon-optimised (DIII-CH3) nucleotidic sequences. **(D)** Plot of the titres from the curves shown in A, B and C.
(TIF)

S3 Fig Conformational ELISA for detection of anti-DIII and anti-sE antibodies. **(A)** Scheme of constructs DIII-BAP (DIII-εCH4-BAP) and sE-BAP-roTag that are secreted from mammalian cells as mono-biotinylated molecules and used in the conformational ELISA. **(B)** Scheme of the ELISA, with avidin-coated plates to capture biotinylated DIII-BAP or sE-BAP-roTag.
(TIF)

S4 Fig Detection of anti-DIII antibodies by cytofluorimetry. **(A)** Scheme of constructs DIII-GPI (DIII-εCH4-GPI) expressed on cell surface membranes. **(B)** Cytofluorimetry of Sp2/0 stably transfected clones displaying the different GPI anchored DIII, detected with the corresponding anti-DIII sera. Ctrl: negative control sera.
(TIF)

S5 Fig DENV4 genotype effect on 4DIII neutralising response. **(A)** Plaque reduction curves on DENV4 TC25 strain using pools of sera from animals vaccinated with 4DIII-CH3 derived from DENV4 Dominica strain (open symbols) or TC25 strain (filled symbols). **(B)** PRNT_50_ titres from curves shown in A
(TIF)
