# Supplementary figures and images for: Dengue E Protein Domain III-Based DNA Immunisation Induces Strong Antibody Responses to All Four Viral Serotypes
Source: PLoS Negl Trop Dis. 2015 Jul 28;9(7):e0003947. doi: 10.1371/journal.pntd.0003947 (PMC4517776; doi:10.1371/journal.pntd.0003947)

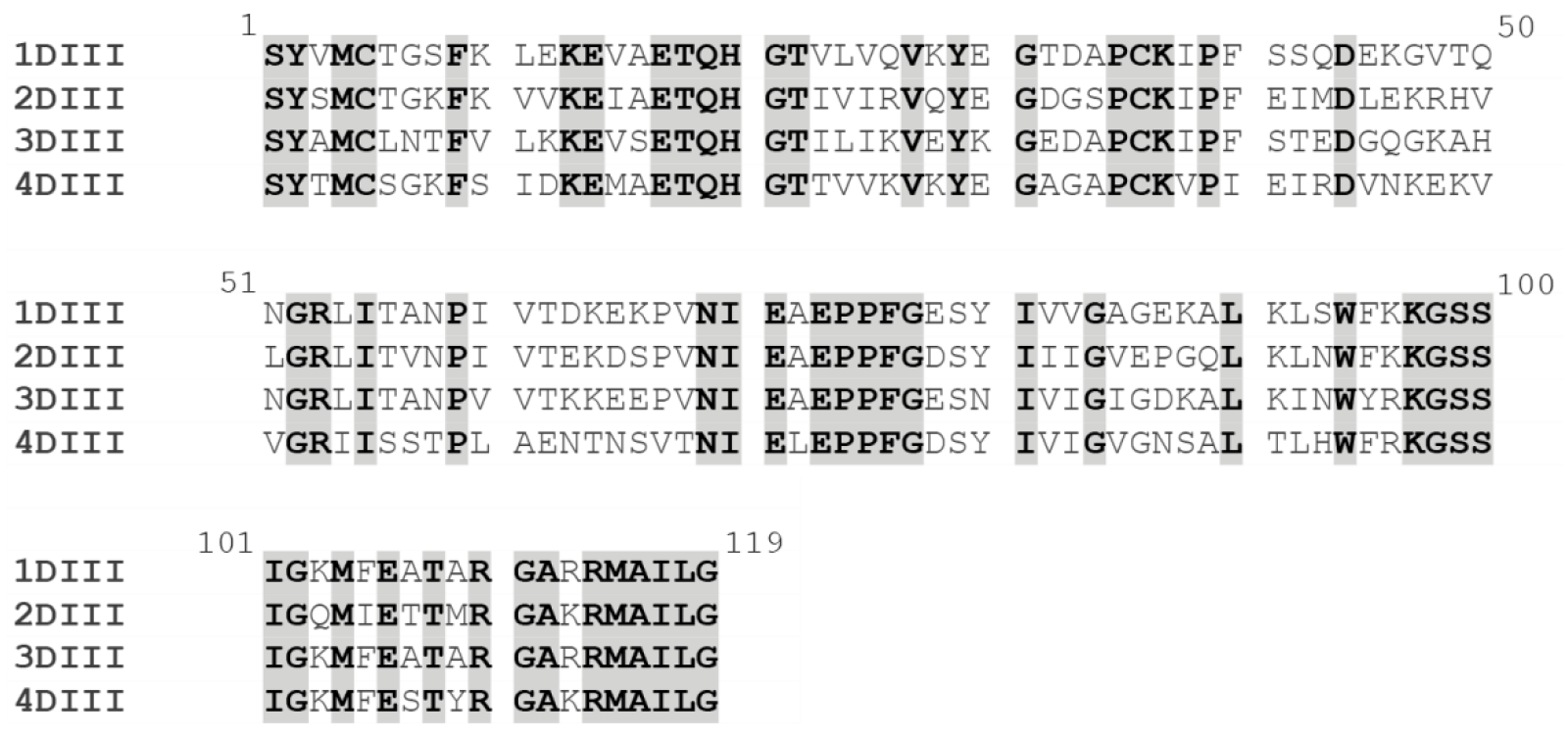

Supplement: S1 Fig — Amino acids conserved across the four serotypes are highlighted. (TIF) [file pntd.0003947.s001.tif]

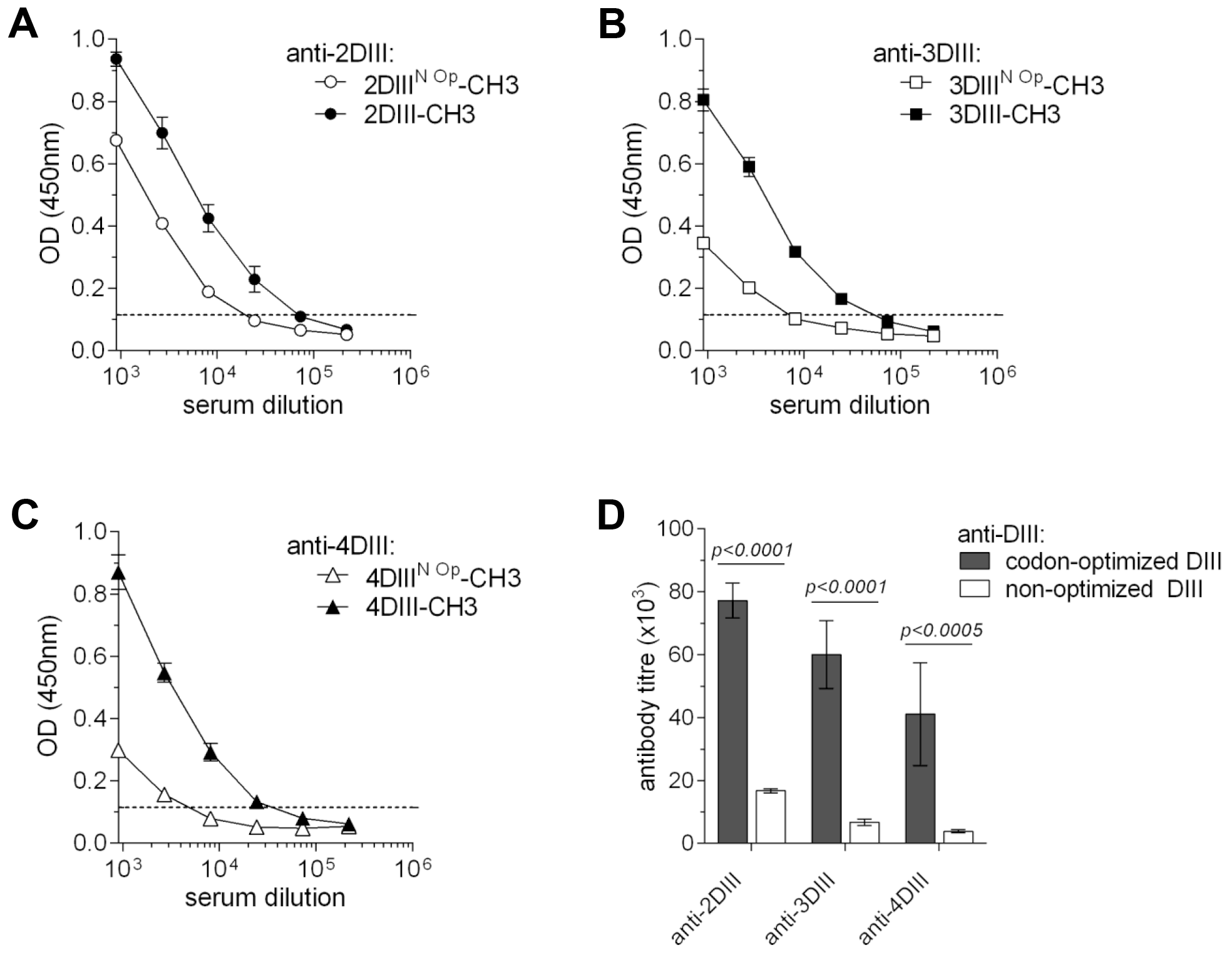

Supplement: S2 Fig — (A-C) ELISA of sera from animals gene-gun immunised with DIII-CH3 constructs from serotypes 2 (A), 3 (B) and 4 (C), with viral (DIIINOp-CH3) or codon-optimised (DIII-CH3) nucleotidic sequences. (D) Plot of the titres from the curves shown in A, B and C. (TIF) [file pntd.0003947.s002.tif]

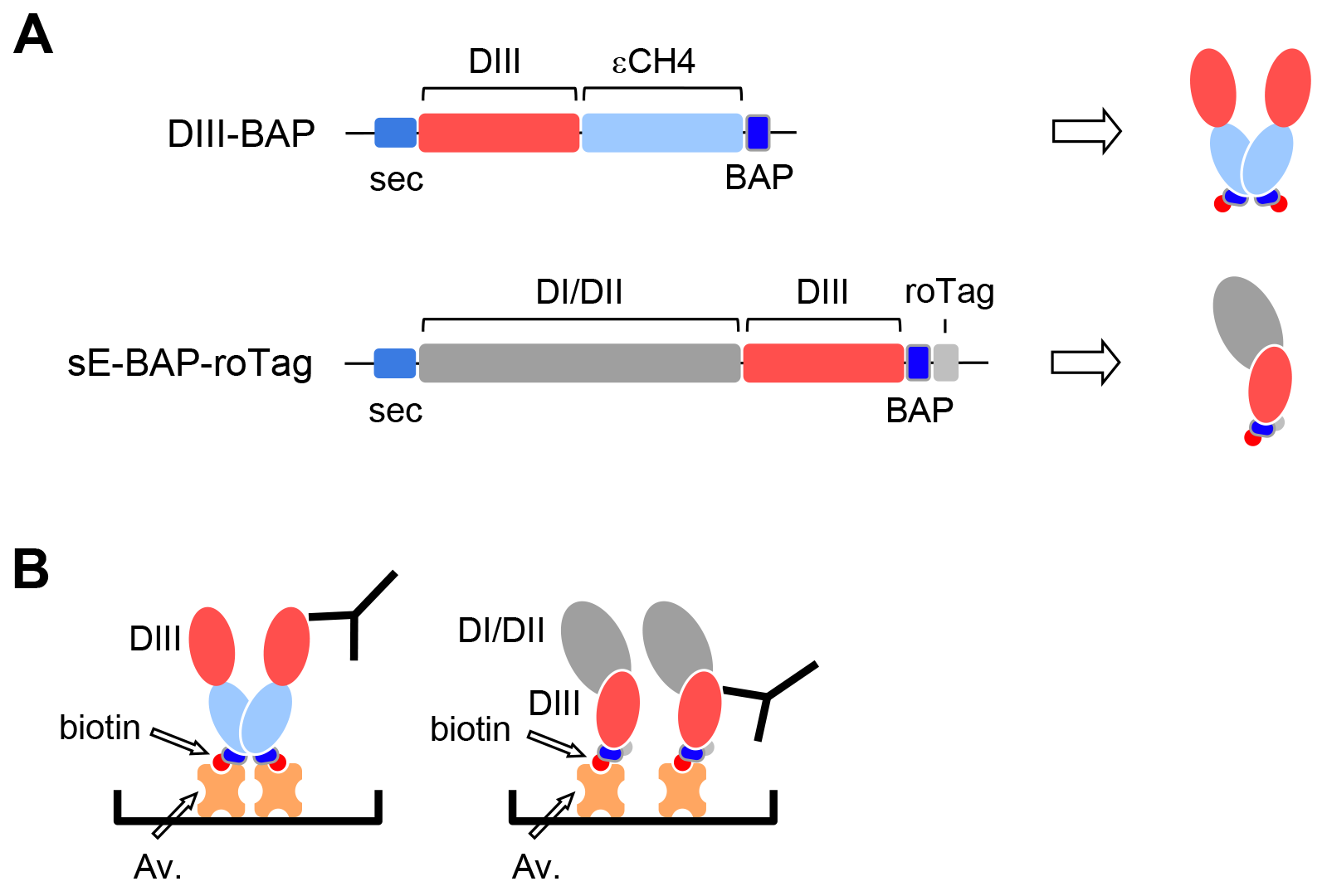

Supplement: S3 Fig — (A) Scheme of constructs DIII-BAP (DIII-εCH4-BAP) and sE-BAP-roTag that are secreted from mammalian cells as mono-biotinylated molecules and used in the conformational ELISA. (B) Scheme of the ELISA, with avidin-coated plates to capture biotinylated DIII-BAP or sE-BAP-roTag. (TIF) [file pntd.0003947.s003.tif]

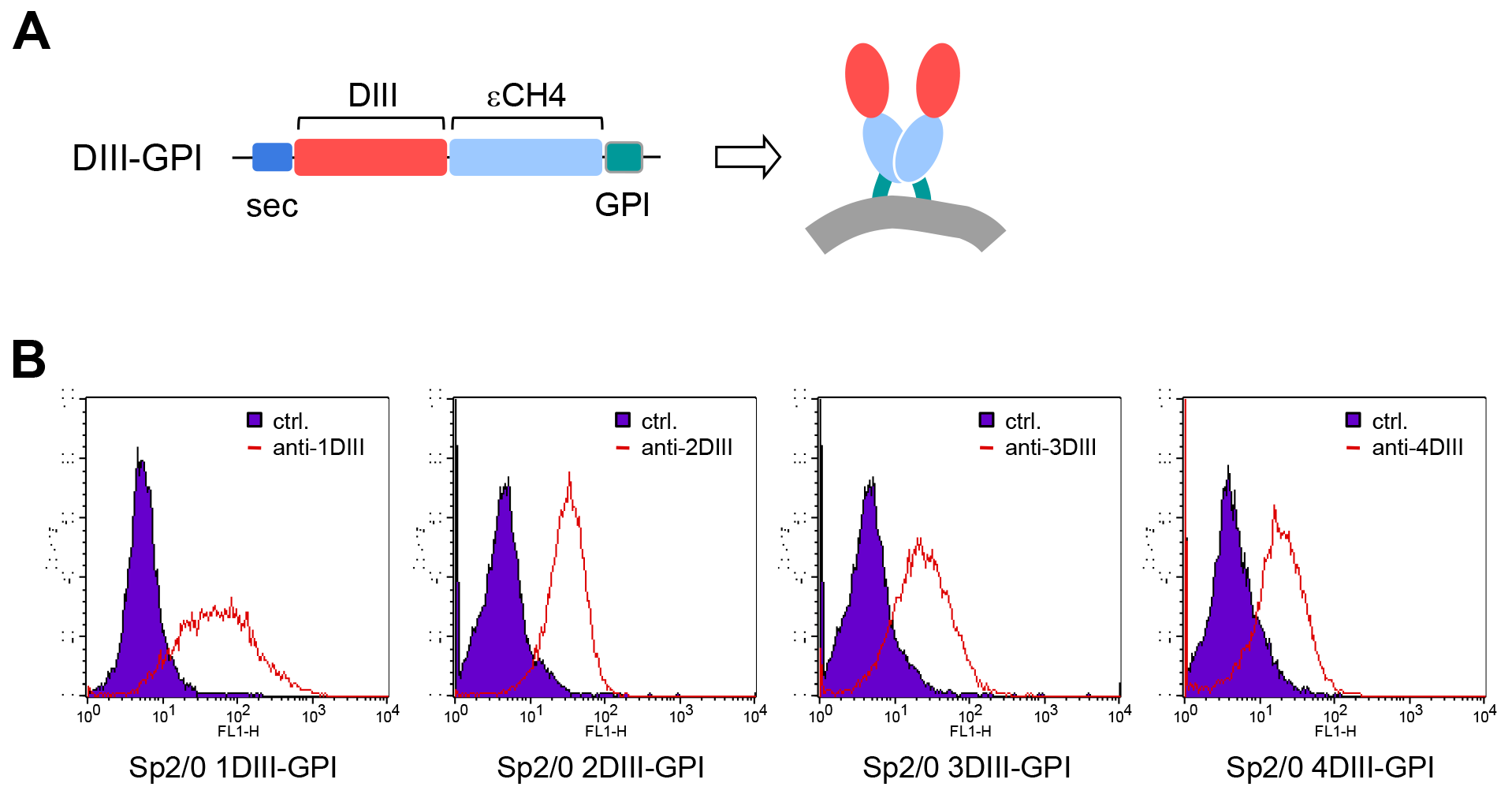

Supplement: S4 Fig — (A) Scheme of constructs DIII-GPI (DIII-εCH4-GPI) expressed on cell surface membranes. (B) Cytofluorimetry of Sp2/0 stably transfected clones displaying the different GPI anchored DIII, detected with the corresponding anti-DIII sera. Ctrl: negative control sera. (TIF) [file pntd.0003947.s004.tif]

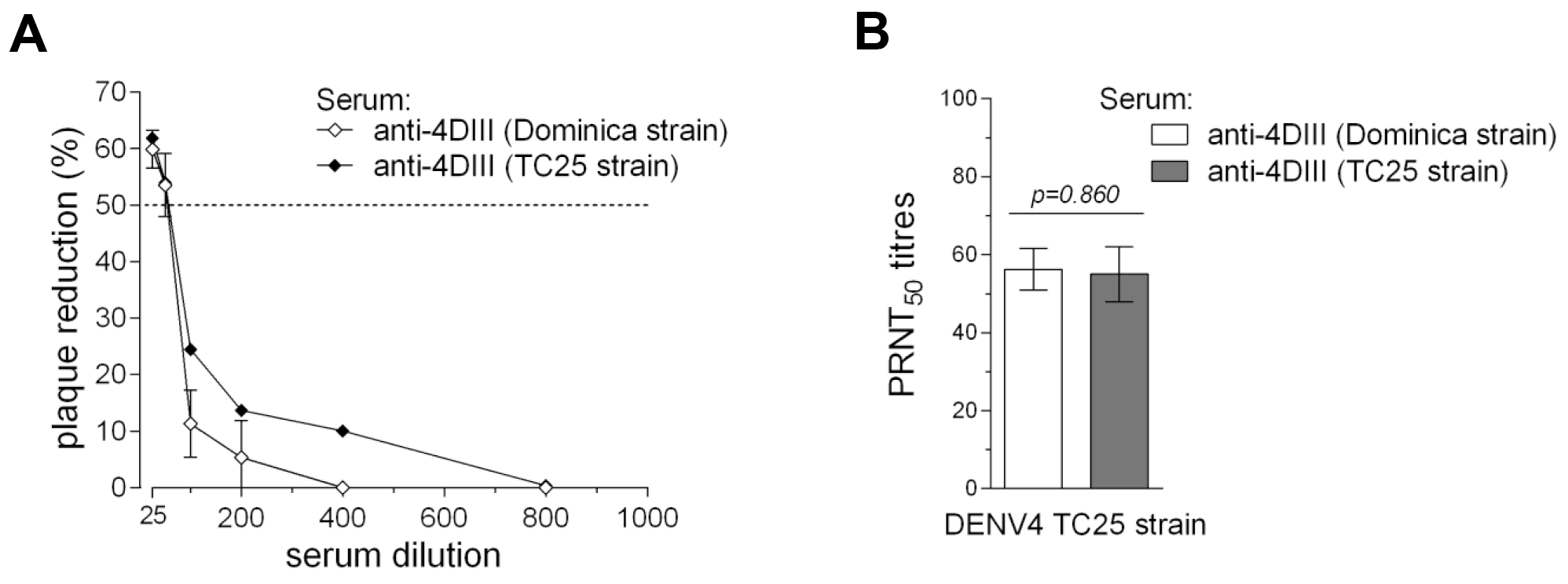

Supplement: S5 Fig — (A) Plaque reduction curves on DENV4 TC25 strain using pools of sera from animals vaccinated with 4DIII-CH3 derived from DENV4 Dominica strain (open symbols) or TC25 strain (filled symbols). (B) PRNT50 titres from curves shown in A (TIF) [file pntd.0003947.s005.tif]
